# 

**Published:** 1854-10

**Authors:** 


					﻿RECEIPTS DURING AUGUST, SEPTEMBER & OCTOBER.
ILLINOIS.
Dr Andrews, 2 00
Lyman, 2 00
W Cromwell 2 00
J E Carr 1 00
W C Walker 4 00
E E Rowers 5 00
W Carpenter 2 00
J M Metcalf 5 00
C Goodbrake 2 '<0
A Mahan 2 00
W P Bjcbard- 2 00
son	2	00
Marr	2	00
J Lyford 2 00
L C White 2 00
It J Stough 2 00
J McHugh 2 00
Il T Goodwin 2 00
A Patterson 3 00
E G Ri-e 6 00
W H Crandall 6 (W
Rouse	2	00
Hamilton 2 00
Cooper	3	CO
Dr Arnold	2	0i>
J S Maue	0	00
TL Fitch	6	00
Welch	2	00
B H Madden	6	00
E L Patten	2	00
E Watkins	G	00
F K Miner	2	00
A B Mead	2	00
Dodson	2	00
Hand & Leroy	2	00
Rogers	2	00
Brothers	4	00
Woodruff	2	00
Trueedail	4	00
Martin	2	00
N L coon	2	00
G V Ewing	2	00
W T Hutchin-
son	4	00
J P Hamilton	2	00
W w Welch	1	50
W II Davis	3	00
J C Brown	0	50
INDIANA.
Dr R Faber 6 00
A T McCurdy 1 00
B F Bussell 2 00
H Dnncan 2 00
A Wood	3	0t>
J Hoover 6 00
Dr TW Miller 2 Oo
S S Cooper 2 00
J S Collings 4 00
A L Pettijohn 4 00
II P Howes 2 00
W II Gifford 2 00
IOWA
Dr'W Reinhart 2 00
J Donor	2 00
Drs Noble & Da-
sbieil	2 00
MICHIGAN
Dr J B W Lewis 2	00
S II Gate	4	00
L Foster	6	00
SheDard	2	00
Dr M Hawn	2 00
W Aij ha	4 01
G S Hunt	6 00
WISCONSIN
Dr Terrell t 4 00
AS Ca.'-t'eman I OO
G "iddell 2 00
E Delauev 2 00
Dr J L Scofield 2 00
111) Rockwood 2 00
K P Wood	2 CO
J W Todd	2 00
Dr E C Still. Mi-souri.	2 CO
.1 R Bradf >ra. California, 1 1*0
W W Hwenev. Min Ter., 5 (0
J B Evans, Ohio,	2 10
				

